# Long noncoding RNA TUG1 facilitates osteogenic differentiation of periodontal ligament stem cells via interacting with Lin28A

**DOI:** 10.1038/s41419-018-0484-2

**Published:** 2018-04-19

**Authors:** Qin He, Shuangyan Yang, Xiuge Gu, Mengying Li, Chunling Wang, Fulan Wei

**Affiliations:** 1 Department of Orthodontics, School of Stomatology, Shandong University, Jinan, People’s Republic of China; 2 Shandong Provincial Key Laboratory of Oral Tissue Regeneration, School of Stomatology, Shandong University, Jinan, People’s Republic of China

## Abstract

Periodontal ligament stem cells (PDLSCs) are mesenchymal stem cells derived from dental tissues with multidirectional differentiation potential and excellent self-renewing ability. Recently, long noncoding RNAs (lncRNAs) have been shown to play important roles in MSC osteogenic differentiation. In this study, we found that taurine upregulated gene 1 (TUG1), an evolutionarily conserved and widely present lncRNA was significantly upregulated in osteogenically induced PDLSCs compared to their undifferentiated counterparts. Further investigation demonstrated that the expression of TUG1 was positively correlated with the osteogenic differentiation of PDLSCs following the induction, as evidenced by the increase in cellular alkaline phosphatase (ALP) level, formation of calcium nodules, and the upregulation of several osteogenic-related gene markers such as ALP, osteocalcin (OCN), and runt-related transcription factor 2 (Runx2). Conversely, TUG1 knockdown was demonstrated to inhibit the potential of PDLSCs for osteogenic differentiation. Using bioinformatics analysis, we identified lin-28 homolog A (Lin28A) as a potential target of TUG1 during osteogenic differentiation of PDLSCs. Lin28A was found to be significantly downregulated in TUG1-repressed PDLSCs and contained multiple binding sites for lncRNA TUG1. Moreover, suppression of Lin28A was shown to be able to inhibit osteogenic differentiation and decreased the expression of several osteogenic genes. Taken together, these results could help researchers better understand the mechanism that governs the osteogenic differentiation of PDLSCs, and also serve as a stepping stone for the development of novel therapeutic strategies that can be used to regenerate dental tissues.

## Introduction

Mesenchymal stem cells (MSCs) have been widely used as basal biomaterials in tissue engineering owing to their multidirectional differentiation potential and ability to self-renew. In stomatology, MSCs derived from orofacial tissues play an important role in tooth development and tissue regeneration. For instance, based on periodontal ligament stem cells (PDLSCs), periodontal tissues, and bioengineered tooth root (bio-root) structure have been successfully regenerated in miniature pigs^1–3^. In this regard, better understanding of the mechanism that governs MSC osteogenic differentiation would greatly facilitate the development of novel therapeutic strategies for tissue regeneration.

Long noncoding RNAs (lncRNAs) are defined as transcripts longer than 200 nucleotides and originally regarded as transcriptional “noise”^4,5^. However, growing evidences indicated that lncRNAs have emerged as key modulators to participate in various physiological and pathological processes, including gene regulation, cell development, tissue formation, and metabolism^6–8^. Many studies on lncRNAs have mainly focused on revealing the biological behaviors and related molecule mechanisms of different cancer cells^9,10^. During normal development processes, there is evidence that the potential roles of lncRNAs could affect a wide range of cellular activities, including cell differentiation, self-renewal, proliferation, apoptosis, etc.^11–13^. In the past decade, rapid development of high-throughput sequencing technologies and bioinformatic analytical methods have enabled researchers to identify lncRNAs that play major roles in the osteogenic differentiation of pluripotent stem cells^14^. For example, Zhu et al.^15^ reported that HoxA-AS3 acted as an epigenetic switch to associate with Enhancer of Zeste 2 (EZH2) and repress the transcription of key osteoblastic factors in bone marrow mesenchymal stem cells (BMSCs). In another study, Cui and coworkers^16^ demonstrated that the knockdown of lncRNA NONHSAT009968 could accelerate the osteogenic differentiation of MSC by inhibiting the expression of staphylococcal protein A. Recently reports have also demonstrated that lncRNA may act as a competing endogenous (ceRNA) for miRNAs, adjusting the expression of their targeting genes in the osteogenic differentiation of MSC^17,18^. In previous study, we found that miR-21 could regulate the osteogenic differentiation of PDLSCs by targeting SMAD family member 5 (Smad5)^19^. With regard to the potential relationship of miRNA and lncRNA, we searched lncRNAs that were correlative to miR-21 and selected three functional lncRNAs for validation. Taurine upregulated gene 1 (TUG1) was one of the related lncRNAs and the expression of TUG1 was most obviously changed in osteogenic differentiated PDLSCs.

LncRNA TUG1, a 7.1 kb lncRNA, was initially identified as an essential gene for retinal development and forming photoreceptors in mouse eye^20^. Recently, TUG1 was considered to be abnormally regulated during tumorigenesis as a potential tumor suppressor or as an oncogene^21,22^. Overexpression of TUG1 was found to be involving in endothelial cell apoptosis, such as atherosclerosis and hepatocellular carcinoma^23,24^. Additional reports indicated that TUG1 can promote the cell proliferation of human non-small cell lung cancer, high-grade muscle-invasive bladder cancer, and esophageal squamous cell carcinoma (ESCC)^25–27^. Further researches have reported that the abnormal expression of TUG1 is associated with the pathogenesis of many neurological disorders^28^. However, the role of TUG1 on the osteogenic differentiation of PDLSCs and its mechanism is still poorly understood.

The purpose of the current study was to investigate whether lncRNAs were mechanistically involved in the osteogenic differentiation of PDLSCs. Our data led to the identification of TUG1 as a likely regulator of PDLSCs osteogenesis. Further studies revealed that TUG1 could promote the osteogenic differentiation of PDLSCs by activating lin-28 homolog A (Lin28A), a RNA-binding protein (RBP). Our results provided novel insights into the mechanism that underlies the osteogenic differentiation of PDLSCs and could serve as a stepping stone for the development of novel therapeutic strategies that can be used to regenerate dental tissues.

## Results

### Identified and induced of PDLSCs osteogenic differentiation

**Fig. 1:**
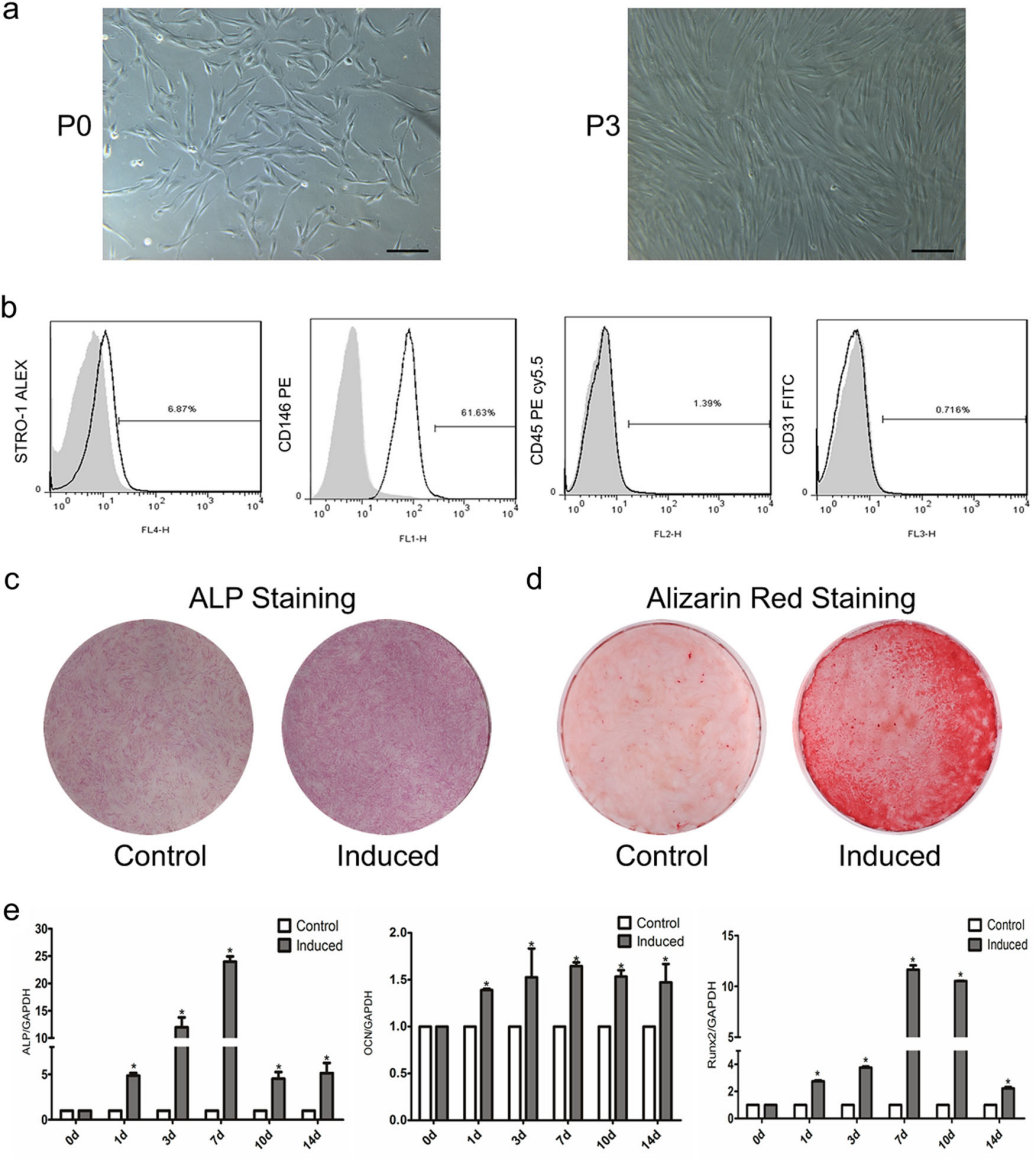
Identification and induction of osteogenic differentiation in human PDLSCs. **a** Imaging of PDLSCs cultured in normal media before the first (P0) or after the third passage (P3) by optical microscopy. **b** The isolated cells expressed mesenchymal stem cell markers including STRO-1 and CD146, but were negative for leucocyte maker (CD45) and platelet endothelial cell marker (CD31). **c**, **d** Osteoblastic differentiation of PDLSCs was induced in an osteogenic-inducing medium as evidenced by ALP staining and Alizarin Red staining. The control group was grown in the standard medium. **e** qRT-PCR analysis of osteogenic marker genes such as ALP, OCN, and Runx2 in osteogenically induced PDLSCs and the non-induced control cells. All experiments were performed in triplicate and results were expressed as means ±SD. *P < 0.05. Scale bar: 200 μm

We began our current study by first assessing the potential of our PDLSCs for osteogenic differentiation. As shown in Fig. 1a, b, cultured PDLSCs successfully exhibited spindle-shaped morphologies and expressed MSC markers (STRO-1 and CD146); but were negative for leucocyte cell maker (CD45) and platelet endothelial cell marker (CD31). Next, cells cultivated in a standard osteogenic induction medium developed an unambiguous osteoblastic phenotype compared to the non-induced control as evidenced by the increasing level of intracellular alkaline phosphatase activity (ALP) and the formation of calcium nodules (Fig. 1c, d). These results were further echoed by the observation of a significant increase in the expression of several osteogenic-related genes, including ALP, osteocalcin (OCN) and runt-related transcription factor 2 (Runx2) up to 14 days following the induction (Fig. 1e). Combined, our data confirmed the ability of our PDLSCs to differentiate into osteoblasts under the osteogenic culturing conditions used in this study.

### Expression of TUG1 was positively correlated with the osteogenic differentiation of PDLSCs

**Fig. 2:**
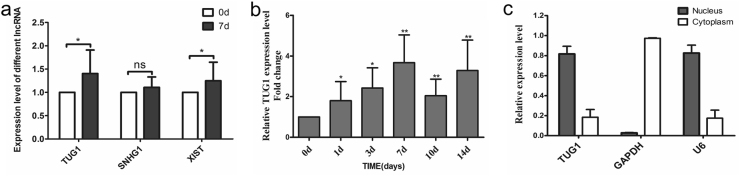
TUG1 expression was positively correlated with PDLSC osteogenic differentiation. **a** The expression levels of lncRNA TUG1, SNHG1, and XIST in PDLSCs cultivated in an osteogenesis-inducing medium for 0 and 7 days. Results at day 0 were used as baselines. **b** Time course of TUG1 expression in PDLSCs at day 0, 1, 3, 7, 10, and 14 after the osteogenic induction as determined by qRT-PCR. **c** Subcellular localization of TUG1 in PDLSCs. The majority of TUG1 was localized in the nucleus rather than cytoplasm. The relative level of the target gene was determined using the comparative threshold cycle (CT) method with GAPDH (cytoplasmic) and U6 (nuclear) as control. All experiments were performed in triplicate and results were expressed as means ±SD. *P < 0.05; **P < 0.01; NS not significant

We have previously reported that miR-21 could play regulatory role in PDLSC osteogenic differentiation^19^. Based on this finding, we sought to continue to explore the possible implication of other noncoding RNAs in the osteoblastic differentiation of PDLSCs. Therefore, we first used StarBase 2.0 to predict the lncRNAs that could potentially interact with miR-21. As summarized in Table 1, we identified a panel of 19 putative lncRNA candidates, among which we selected three functional lncRNAs that have been previously reported, including TUG1, Small nucleolar RNA host gene 1 (SNHG1) and X inactive-specific transcript (XIST). Quantitative real-time reverse transcription polymerase chain reaction (qRT-PCR) analysis of the expression levels of the three genes mentioned above indicated that TUG1 and XIST were both significantly upregulated in osteoblastic differentiated PDLSCs compared to the non-differentiated control group on day 7 after the induction (Fig. 2a). We then selected TUG1 for further study because there has been ample evidence for the regulatory role of XIST in the pluripotent differentiation of various stem cells^29–32^, whereas the role of TUG1 is less explored. We measured the level of TUG1 in PDLSCs cultured in an osteogenic-inducing medium at different time points. The results indicated that TUG1 expression was upregulated in a time-dependent manner following the induction of osteogenic differentiation in PDLSCs (Fig. 2b). Further quantitative analysis of TUG1 expression in the cytoplasmic and nuclear RNA isolated from non-differentiated PDLSCs suggested that the lncRNA was broadly localized in the cells but showed a significantly higher level in the nucleus than cytoplasm (*P* < 0.05, Fig. 2c), which echoed the results in a previously published study^33,34^.

**Table 1:** Bioinformatics predicting lncRNAs associated with miRNA21

Name	Mir accession	Gene name
hsa-miR-21-5p	MIMAT0000076	TUG1
hsa-miR-21-5p	MIMAT0000076	SNHG1
hsa-miR-21-5p	MIMAT0000076	XIST
hsa-miR-21-5p	MIMAT0000076	AL589743.1
hsa-miR-21-5p	MIMAT0000076	RP11-869B15.1
hsa-miR-21-5p	MIMAT0000076	CTC-228N24.3
hsa-miR-21-5p	MIMAT0000076	RP11-282O18.3
hsa-miR-21-5p	MIMAT0000076	AC000120.7
hsa-miR-21-5p	MIMAT0000076	RP11-488I20.9
hsa-miR-21-5p	MIMAT0000076	RP11-834C11.4
hsa-miR-21-5p	MIMAT0000076	C11orf95
hsa-miR-21-5p	MIMAT0000076	RP11-498D10.6
hsa-miR-21-5p	MIMAT0000076	GS1-251I9.4
hsa-miR-21-5p	MIMAT0000076	CTC-241F20.3
hsa-miR-21-5p	MIMAT0000076	FAM201A
hsa-miR-21-5p	MIMAT0000076	KB-1615E4.2
hsa-miR-21-5p	MIMAT0000076	AC025171.1
hsa-miR-21-5p	MIMAT0000076	AL163636.6
hsa-miR-21-5p	MIMAT0000076	RP11-293M10.6

### Downregulation of TUG1 inhibited the osteogenic differentiation of PDLSCs

**Fig. 3:**
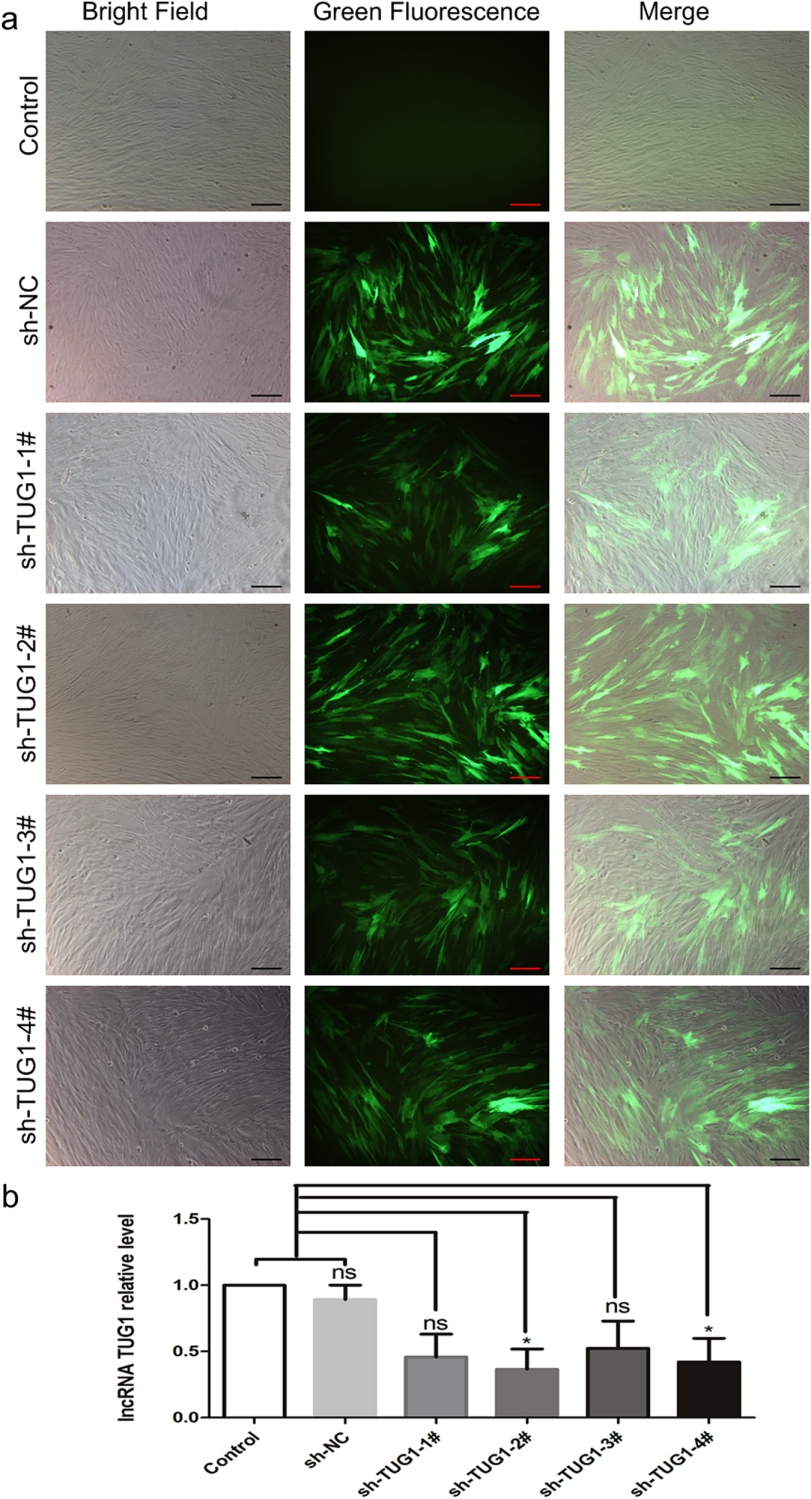
TUG1 knockdown in PDLSCs by lentiviral transfection. sh-TUG1-1# to sh-TUG1-4# represented four lentiviral constructs containing small hairpin inserts based on different regions of TUG1 sequence. Fluorescence imaging (**a**) and qRT-PCR analysis (**b**) were performed 72 h after transfection and both found sh-TUG1-2# to have the most pronounced downregulating effect. All experiments were performed in triplicate and results were expressed as means ±SD. *P < 0.05; NS not significant. Scale bar: 200 μm

The regulatory effect of TUG1 on PDLSC osteogenesis was further probed by gene knockdown experiments using a series of lentiviral constructs, each encoding a short hairpin RNA that corresponded to a specific region of the lncRNA (Table 2). After verification of successful transfection by fluorescence imaging (Fig. 3a), the expression level of TUG1 in PDLSCs treated with different lentiviral constructs were determined. As shown in Fig. 3b, the results demonstrated that PDLSCs transfected with sh-TUG1-2# showed the lowest level of endogenous TUG1 expression (*P*-value: sh-TUG1-1# = 0.052, sh-TUG1-2# = 0.025, sh-TUG1-3# = 0.104, sh-TUG1-4# = 0.048, respectively). Thus, sh-TUG1-2# was selected as the most effective lentiviral construct for TUG1 knockdown in PDLSCs, for further experiments.

**Table 2:** The interfering sequences for sh-TUG1 and sh-NC

Gene name	Sequence
sh-NC	5′-TTCTCCGGAACGTGTCACGT-3′
sh-TUG1-1#	5′-CTGTTGACGCTTGCTGTGAGAA-3′
sh-TUG1-2#	5′-GCTTGGCTTCTATTCTGAATCCTTT-3′
sh-TUG1-3#	5′-GCTACAACTTATCTTCCTTTAC-3′
sh-TUG1-4#	5′-GCAAGAGAATAACTATGAAAGC-3′

**Fig. 4:**
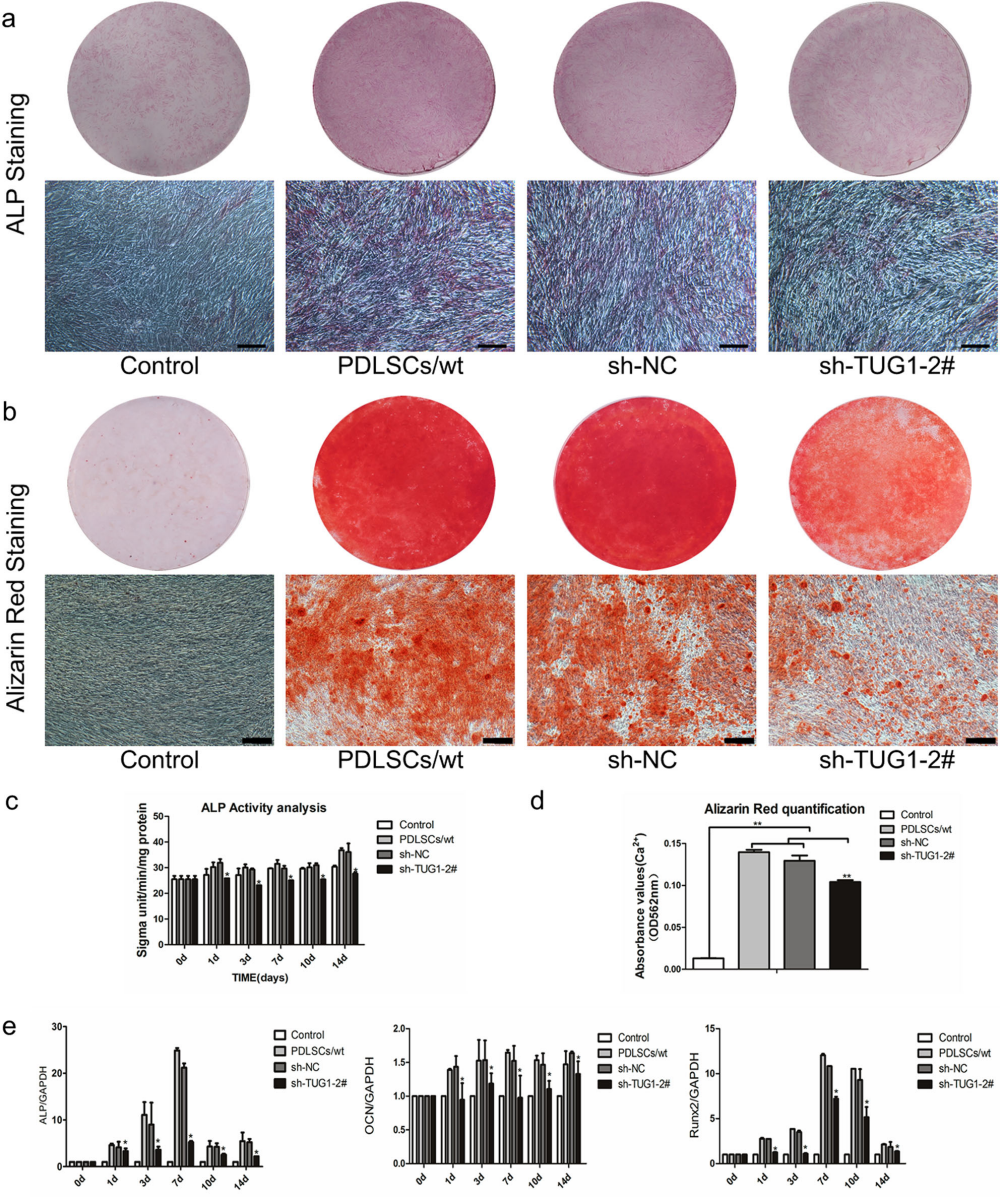
Effect of TUG1 knockdown on the osteogenic differentiation of PDLSCs. Four experiment groups were set up, including the control group, the PDLSCs/wt group, the sh-NC group, and the sh-TUG1-2# group. The sh-TUG1-2# group and the sh-NC group were transfected with the lentiviral constructs encoding specific hairpin RNA, respectively, whereas both the PDLSCs/wt group and the control group were not subjected to transfect. In addition, except for the control group, which was cultured in a standard medium, all the other experiment groups were grown in an osteogenesis-inducing medium. ALP staining (**a**) and Alizarin Red staining (**b**) were performed at the day 7 and 21 after the osteogenic induction on all four experiment groups. Quantification of ALP activity (**c**) and cellular calcium level (**d**) in all experiment groups were measured at different time points after the induction. **e** qRT-PCR analysis of ALP, OCN, and Runx2 mRNA levels in different experiment groups at day 0, 1, 3, 7, 10, and 14 after the osteogenic induction. All experiments were performed in triplicate and results were expressed as means ±SD. *P < 0.05; **P < 0.01. Scale bar: 200 μm

We next examined whether suppressed TUG1 expression could lead to diminished potential of PDLSCs osteogenic differentiation. We set up four experimental groups, including the control group, PDLSCs/wt group, sh-NC group, and sh-TUG1-2# group. The sh-TUG1-2# group and the sh-NC group were transfected with sh-TUG1-2# and a control lentiviral construct sh-NC encoding a nonspecific hairpin RNA, respectively, whereas both the PDLSCs/wt group and the control group were not subjected to transfection. In addition, except for the control group, which was cultured in a standard medium, all the other experiment groups were grown in an osteogenic-inducing medium. Both ALP staining and ALP activity analysis detected a significant decrease in ALP expression in the sh-TUG1-2# group compared to the other three groups (Fig. 4a, c). Similarly, Alizarin Red staining and the measurement of calcium levels also found calcium deposition in induced PDLSCs to be markedly reduced by the knockdown of TUG1 (Fig. 4b, d). These results were further supported by the qRT-PCR analysis of ALP, Runx2, and OCN. The expressions of ALP, OCN, and Runx2 were significantly reduced in the knockdown group than in the sh-NC group or un-transfected cells after induction (*P* < 0.05) (Fig. 4e). Altogether, these results strongly suggested that TUG1 was involved in regulating the osteogenic differentiation of PDLSCs.

### TUG1 and Lin28A positively regulate each other during PDLSCs osteogenic differentiation

**Fig. 5:**
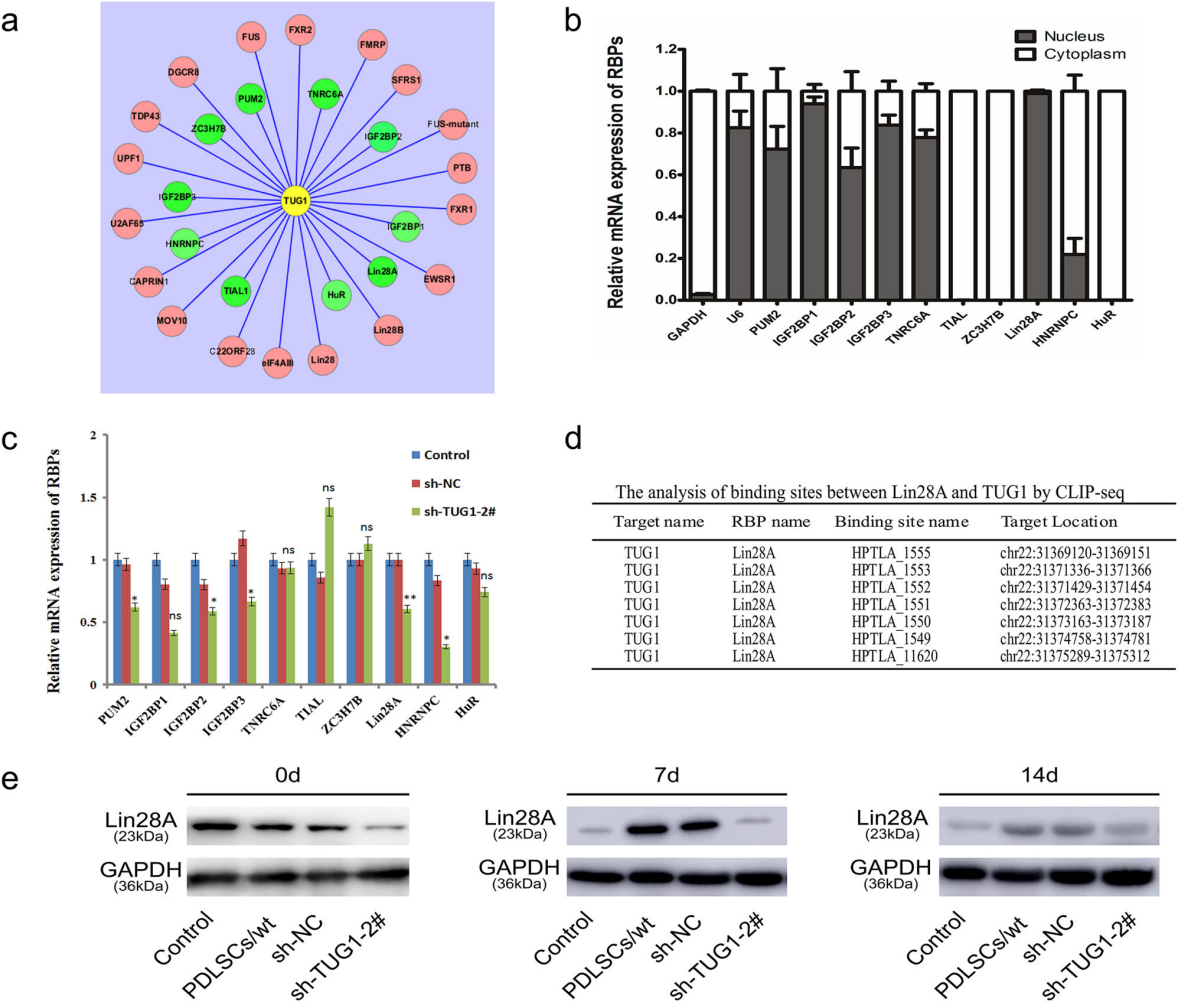
Identification and validation of potential RBPs of TUG1. **a** An interaction network map showing 28 putative RBP candidates that could potentially bind to TUG1. A total of ten candidates were selected for further validation based on literature search, which were shown in green. **b** Subcellular localization of ten RBPs in TUG1 knockdown PDLSCs as determined by qRT-PCR measurement of nuclear and cytoplasmic RNA. GAPDH is the positive control for cytoplasm and U6 is the positive control for nucleus. **c** qRT-PCR analysis of the gene expression levels for the selected RBPs in TUG1 knockdown PDLSCs. **d** Summary of putative binding sites on Lin28A for TUG1 based on results generated from gene co-expression network and CLIP analysis. **e** Western blotting analysis of Lin28A levels in the four above mentioned experiment groups at day 0, 7, and 14 after the osteogenic induction. All experiments were performed in triplicate and results were expressed as means ±SD. *P < 0.05; **P < 0.01; NS not significant

There is increasing evidence that RBPs could mediate the ability of lncRNAs to regulate gene expression^35,36^. To investigate whether RBPs could also be involved in TUG1-mediated osteogenic differentiation of PDLSCs, we scanned Starbase for potential target proteins of TUG1 and identified 28 putative candidates from different cell types (see in Table 3 and Fig. 5a). We then focused on ten RBPs that have been previously reported to play regulatory roles in the differentiation of stem cells. qRT-PCR analysis indicated that the mRNA transcripts of PUM2, IGF2BP1, IGF2BP2, IGF2BP3, Lin28A, and TNRC6A were preferentially localized in the nucleus (Fig. 5b) and showed differential expression levels between TUG1-repressed PDLSCs and untreated or control RNA-transfected cells (Fig. 5c). Among these candidates, Lin28A showed the highest transcriptional level in the nucleus and thus was selected for further analysis. Crosslinking-immunoprecipitation and high-throughput sequencing (CLIP-seq) analysis revealed that Lin28A contained multiple putative binding sites for TUG1, one of which, HGGAGWA, was further confirmed by RBPmap (Fig. 5d and supplementary material 1). Gene co-expression network analysis indicated that the interaction between Lin28A and TUG1 would be thermodynamically favorable with its hybridization energy at −11.8782 kcal/mol (supplementary material 2). Furthermore, western blotting found that the protein level of Lin28A declined significantly in TUG1-repressed PDLSCs compared to the un-transfected cells or those treated with the control lentiviral construct (Fig. 5e). Taken together, these results suggested a correlation between the level of TUG1 and that of Lin28A during the osteogenic differentiation of PDLSCs.

**Table 3:** Bioinformatics predicting RNA-binding proteins associated with lncRNA TUG1

Gene name	Name	Ensembl (human)	Target sites
TUG1	PUM2	ENSG00000055917	2
TUG1	TNRC6A	ENSG00000090905	3
TUG1	FMRP	ENSG00000114416	142
TUG1	SFRS1	ENSG00000136450	1
TUG1	ZC3H7B	ENSG00000100403	22
TUG1	DGCR8	ENSG00000128191	8
TUG1	FXR2	ENSG00000129245	4
TUG1	FUS	ENSG00000089280	14
TUG1	FUS-mutant	ENSG00000089280	1
TUG1	UPF1	ENSG00000005007	340
TUG1	TDP43	ENSG00000120948	1
TUG1	PTB	ENST00000356948	22
TUG1	IGF2BP2	ENSG00000073792	39
TUG1	FXR1	ENSG00000114416	7
TUG1	U2AF65	ENSG00000063244	67
TUG1	HNRNPC	ENSG00000092199	26
TUG1	eIF4AIII	ENSG00000141543	59
TUG1	Lin28B	ENSG00000187772	5
TUG1	EWSR1	ENSG00000182944	8
TUG1	IGF2BP3	ENSG00000136231	33
TUG1	MOV10	ENSG00000155363	16
TUG1	HuR	ENSG00000066044	24
TUG1	IGF2BP1	ENSG00000159217	35
TUG1	Lin28A	ENSG00000131914	7
TUG1	Lin28	ENSG00000131914	10
TUG1	CAPRIN1	ENSG00000135387	3
TUG1	TIAL1	ENSG00000151923	3
TUG1	C22ORF28	ENSG00000100220	4

### Knockdown of Lin28A suppressed the osteogenic differentiation of PDLSCs

**Fig. 6:**
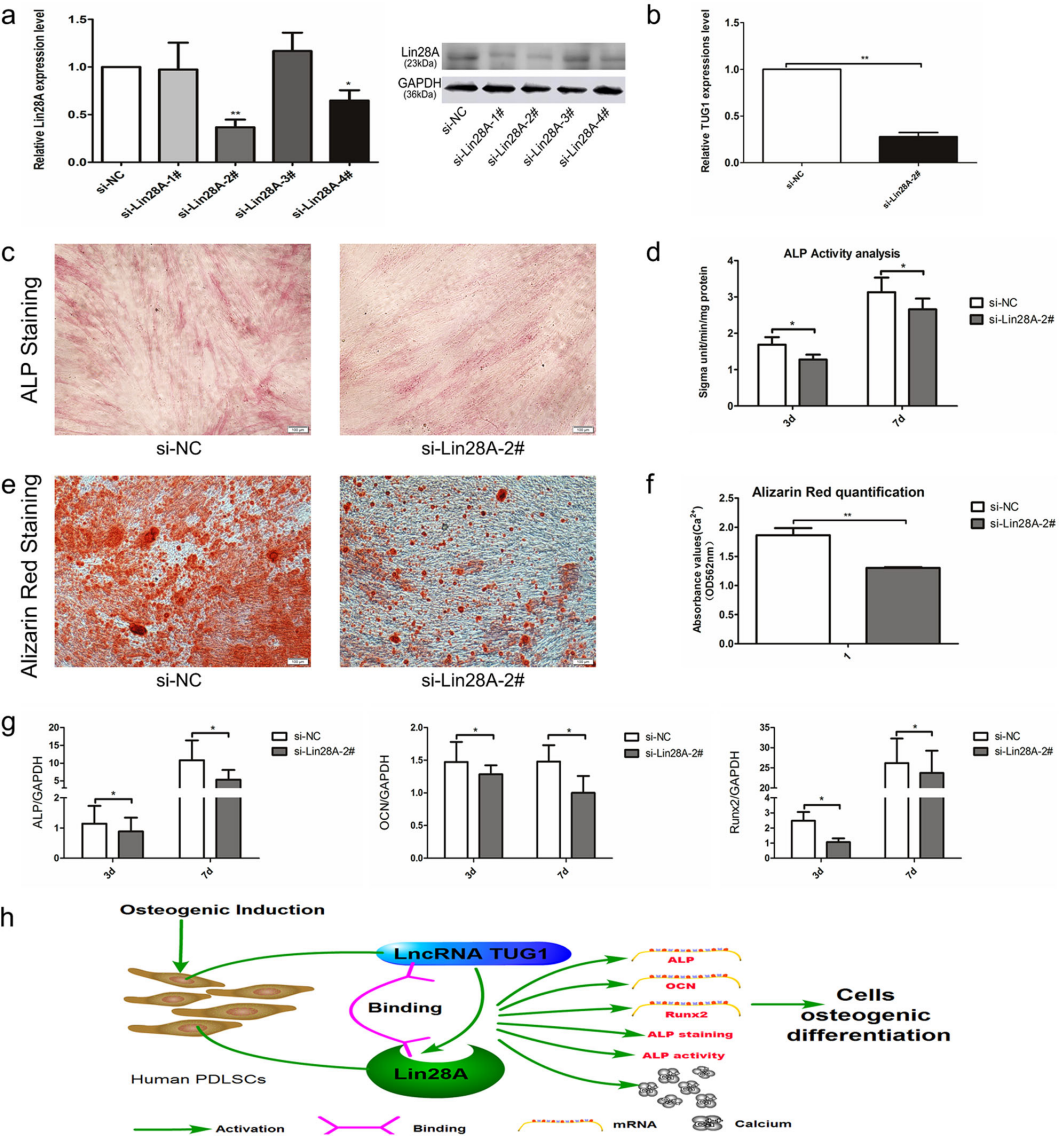
Effects of Lin28A knockdown on the osteogenic differentiation of PDLSCs. **a** Transfection effects of small interference RNAs of Lin28A (si-Lin28A) were determined by qPCR and western blot. **b** TUG1 expression was measured by qPCR in PDLSCs transfected with si-Lin28A-2#. **c**–**g** The functions of siLin28A on the expression of key osteogenic markers of PDLSCs were measured by ALP staining, ALP activity, Alizarin Red staining, and qPCR after inducing different times. ALP staining (**c**) and Alizarin Red staining (**e**) were performed at the day 7 and 21 after the osteogenic induction on all four experiment groups. Quantification of ALP activity (**d**) and cellular calcium level (**f**) in all experiment groups were measured at different time points after the induction. **g** qRT-PCR analysis of ALP, OCN, and Runx2 mRNA levels in different experiment groups at day 0, 1, 3, 7, 10, and 14 after the osteogenic induction. **h** An illustrative view of the TUG1-Lin28A network and its role in the regulation of PDLSC osteogenesis. All experiments were performed in triplicate and results were expressed as means ±SD. *P < 0.05; **P < 0.01. Scale bar: 100 μm

The role of Lin28A in the osteogenic differentiation of PDLSCs was further evaluated by gene knockdown experiments using small interfering RNA (siRNA). Similar to the silencing of TUG1 described above, four siRNA oligonucleotides complementing with different regions of Lin28A were synthesized and transfected individually into PDLSCs (Table 4). Both qRT-PCR and western blotting found si-Lin28A-2# to be the most effective suppressor of Lin28A (Fig. 6a). Subsequent knockdown of Lin28A with si-Lin28A-2# not only led to a significant reduction in the expression level of TUG1 (Fig. 6b), but was also shown to lower the osteogenic potential of the transfected PDLSCs, as evidenced by the decreased ALP staining and ALP activity (Fig. 6c, d). Consistently, both Alizarin Red staining and quantitation of calcium with cetylpyridinium chloride indicated that diminished Lin28A expression could hinder the formation of mineral nodes (Fig. 6e, f). Furthermore, knockdown of Lin28A was found to downregulate osteogenesis markers, such as ALP, OCN, and Runx2, between day 3 and 7 after the induction (Fig. 6g). Taken together, these data implied that Lin28A could be implicated in the modulation of osteogenic differentiation in PDLSCs.

**Table 4:** The small interfering sequences of si-Lin28A and si-NC

Gene name	Sequence
si-NC	Forward primer 5′-UUCUCCGAGACGUGUCACGUTT-3′
	Reverse primer 5′-ACGUGACCACGUUCGGAGAATT-3′
si-Lin28A-1#	Forward primer 5′-CGGGACAGAAUGCAAUAGAATT-3′
	Reverse primer 5′-UUCUAUUUGCAUUUGUCCCGTT-3′
si-Lin28A-2#	Forward primer 5′-CGCUGUGAAGAUCACCGCAATT-3′
	Reverse primer 5′-UUGCGGUGGAUCUCACAGCGTT-3′
si-Lin28A-3#	Forward primer 5′-CCAGAUUCAGGUUAGGCCUATT-3′
	Reverse primer 5′-UAGGCCUCAACCUAAUCUGGTT-3′
si-Lin28A-4#	Forward primer 5′-AAGACUUTAUUGGUACGCAATT-3′
	Reverse primer 5′-UUGCGUAACCAAUAAGUCUUTT-3′

## Discussion

A number of recent studies have suggested a possible regulatory role of lncRNAs in the osteogenic differentiation of pluripotent stem cells. For example, Huang et al.^37^ showed that the ability of H19 to augment the osteogenic capacity of MSCs through the transforming growth factor-β1/Smad family member 3/histone deacetylase signaling pathway. Another study conducted by Jin et al.^38^ reported lncRNA MIR31HG could shift the differentiation potential of human adipose-derived MSCs from adipogenic to osteogenic lineage by activating IkBa and through with the NF-kB pathway. Similarly, there has been convincing evidence that lncRNAs were also involved in the modulation of PDLSC osteogenic differentiation^39^. Downregulation of lncRNA ANCR was found to promote the proliferation and osteogenic differentiation of PDLSCs through activating canonical WNT signaling pathway^40^. Wang and colleagues^18^ demonstrated that LncRNA-POIR could abolish the repression of FoxO1 by miR-182 and concomitantly increase bone formation in PDLSCs from periodontitis patients. Combined with these data, we found that fully understanding the function of target lncRNA in PDLSCs osteogenic differentiation could be helpful to reveal the mechanism of local bone formation. Therefore, based on our previous studies on miR-21, we identified a new lncRNA, TUG1, that could be implicated in regulating the osteogenic differentiation of PDLSCs.

TUG1 was a highly conserved lncRNA capable of regulating a wide range of pathophysiological processes. Subsequent research revealed that dysregulation of TUG1 had been associated with multiple human cancers^41–44^. Emerging studies also show that TUG1 could act as an endogenous sponge to silence miRNAs expression and regulate the activity of target genes. For example, TUG1 has been reported to stimulate cellular growth, migration and epithelial mesenchymal transition (EMT) by sponging miR-382 and interacting with EZH2 in pancreatic cancer^45^. Wang et al.^46^ expounded that TUG1 contain miR-335-5p responsive elements and increased Rho-associated coiled-coil-containing protein kinase 1 expression to strengthen the capacity of cell migration and invasion in osteosarcoma. Cai et al.^47^ illuminated that TUG1 as a competitor of miR-299 could promote the expression of vascular endothelial growth factor A and accelerate tumor-induced angiogenesis in human glioblastoma. Despite these findings, the substantial impact of TUG1 on the osteogenic differentiation of PDLSCs, has not been determined. In the present study, we first observed that TUG1 was mainly distributed in the nucleus of PDLSCs and the dynamic expression of TUG1 was increased in osteogenic-induced PDLSCs. Our silencing experiments also confirmed that reducing TUG1 expression led to a marked inhibition of PDLSC osteogenesis. Interestingly, the connection between TUG1 upregulation and augmented osteogenesis was also noted by Yu et al. in human aortic valves^48^. In the study, the authors found knockdown of miR-204-5p to be able to reverse the TUG1-promoted suppression of Runx2 and stimulation of osteogenic differentiation. This, combined with our current findings, implied that the mechanism through which TUG1 induces osteogenesis could involve multiple pathways or vary in different cell types.

There has been mounting evidence that lncRNAs exert their regulatory functions often via interacting with DNA, RNA, or proteins^49^. From the epigenetic point of view, some specific lncRNAs can function as coordinator of chromatin modification to recruit protein complexes and change DNA methylation status, and thus control the expression of related genes^50^. According to the interaction with miRNAs, many lncRNAs could show their miRNA sponge-like potential and competed for miRNA binding sites to affect the targeting factors activities in various biological processes^51,52^. In addition, lncRNAs can bind to specific protein, thereby affecting the functional of this protein in the process of cellular development. RBP, as a new type of proteins that influences the regulatory function of lncRNA, also receives the special attention of researchers^53–55^. Kim et al.^56^ reported that lncRNA OIP5-AS1 could bind to HuR and inhibit its effect on promoting the proliferation of HeLa cells. Smith et al.^11^ demonstrated that lncRNA Cyrano as a sponge of RBPs restrains the behavior of miR-7 and maintains embryonic stem cell (ESC) in its initial state. Although there are few reports of detailed studies on lncRNA and RBP, it also suggests that some unexplored mechanisms may be hidden between these two important types of molecules. In the current study, we demonstrated that the expression of Lin28A was significantly decreased on both an mRNA and a protein level in TUG1-silenced PDLSCs. Further bioinformatic analysis suggested that Lin28A contained multiple binding sites for TUG1 and the putative interaction between the two would be thermodynamically favorable. Therefore, our experiment data showed a clear correlation between the level of TUG1 and that of Lin28A during the osteogenic differentiation of PDLSCs, though further investigation is needed to ascertain whether the two could really interact with each other.

Lin28A, first identified in *Caenorhabditis elegans*, has been shown to be a key reprogramming factor to participate in cellular development, metabolism and stem cell maintenance^57,58^. Daley et al.^59^ reported that Lin28A as an essential coordinator blocks miRNA-mediated differentiation to maintain somatic cells in their embryonic state. Li et al.^60^ confirmed that Lin28A has uniqueness binding sites of specific lncRNAs, the combination of which may coordinately determine multiple cellular activities in human cancer and genetic diseases. Moreover, Lin28A and Eprn (a lncRNA) constituted a multipronged network to modulate mouse ESCs pluripotency^61^. Our silencing experiment strongly suggested that Lin28A and TUG1 existed a synergistic regulation in PDLSCs; then emerged a decrease tendency of several osteogenic differentiation markers when inhibiting Lin28A expression. Comprehensive understanding of these findings, we realized that Lin28A could be a downstream target of TUG1 and might play an important role in the induction of PDLSC osteogenesis.

In summary, our study showed that upregulation of TUG1 was correlated with the osteogenic differentiation of PDLSCs. We also demonstrated that a connection between the expression level of Lin28A and that of TUG1 in osteogenically differentiated PDLSCs. We believe that TUG1, as an important osteogenesis regulator, could be used for periodontal tissue engineering and guiding bone regeneration.

## Materials and methods

### Chemicals

All chemicals, biological reagents, and kits were purchased from Sigma-Aldrich (St. Louis, MO, USA) unless specified otherwise.

### PDLSCs isolation, culture, and osteogenic differentiation

All experiments in this study were approved by the Medical Ethical Committee of School of Stomatology, Shandong University (No. G201401601). Our dental samples consisted of intact first or second premolars extracted from teenagers aged between 10 and 16 years old under their and their parents’ informed consent. Periodontal ligament tissues were isolated from the teeth as previously described^2,19^. Briefly, the tissues were gently separated from the middle third of dental roots and digested in alpha modification of Eagle’s medium (α-MEM, HyClone, South Logan, UT, USA) supplemented with 3 mg/ml of collagenase type I and 4 mg/ml of dispase II for 0.5 h at 37 °C. The resultant cell suspension was subsequently passed through a 70 μm strainer (Biologix, USA) and centrifuged at 1000×*g* for 5 min. The cell pellet was isolated and transferred to 25 cm^2^ culture flasks containing α-MEM supplemented with 20% fetal bovine serum (FBS, Gibco, Invitrogen, Carlsbad, CA, USA), 2 mmol/l of glutamine, 100 U/ml of penicillin, and 100 μg/ml streptomycin (Invitrogen, Carlsbad, CA, USA). Cell culture was performed at 37 °C in 5% carbon dioxide. The stem cell properties of PDLSCs were characterized by cell surface markers (STRO-1, CD146, CD31, and CD45) at flow cytometer (Becton, Franklin Lakes, NJ, USA). The culture medium was changed every 2–3 days until between the third and the sixth passage. To detect osteogenic differentiation, cells were seeded into the six-well culture plates at 60–70% confluency and induced after reaching 70–80% confluency by using an osteogenic-inducing medium consisting of α-MEM supplemented with 10% FBS, 50 μg/ml of vitamin C sodium salt, 10 mmol/l β-glycerophosphate disodium salt hydrate and 0.1 μmol/l dexamethasone. A control group was set up in parallel where the PDLSCs were cultivated in α-MEM supplemented with 10% FBS under otherwise identical conditions. In each case, the culture medium was changed every 2 or 3 days.

### ALP staining and ALP activity assay

PDLSCs were induced in the osteogenic-inducing medium. The level of ALP activity in the cells was measured using an ALP Activity Kit at day 0, 1, 3, 7, 10, and 14 after the induction. In addition, cellular ALP was also visualized at day 7 after the induction by fixing the cells with 4% paraformaldehyde, followed by staining with a solution of 0.25% naphthol AS-BI phosphate and 0.75% fast red violet provided in the Leukocyte ALP Kit.

### Alizarin Red staining and quantification

PDLSCs were induced in the osteogenic-inducing medium for 21 days, fixed with 70% ethanol, stained with 2% Alizarin Red (pH = 4.24) and imaged under an inverted microscope. To measure the concentration of calcium, the Alizarin Red dye in the PDLSCs was extracted with 400 µl of 10% (w/v) cetylpyridinium chloride in 10 mM sodium phosphate solution for 10 min at room temperature, and then quantified on a UV–Vis spectrometer at 562 nm.

### qRT-PCR

Total RNA extraction was performed using the Trizol Reagent according to the manufacturer’s protocol. Next, 1 μg of the extracted RNA was reverse-transcribed by using PrimeScript RT reagent Kit with gDNA Eraser (Takara, Japan). The obtained cDNA was used as template for subsequent qRT-PCR reactions on a LightCycler480 II Real-Time PCR System (Roche Diagnostics, Switzerland) using the following cycling parameters: 95 °C for 30 s, followed by 45 cycles of 95 °C for 5 s, 60 °C for 35 s, and 50 °C for 30 s. The relative level of the target gene was determined using the comparative threshold cycle (CT) method with glyceraldehyde-3-phosphate dehydrogenase (GAPDH) as control. Primer sequences were summarized in Table 5.

**Table 5:** qRT-PCR Primer sequence

Gene name	Sequence
Human TUG1	Forward primer 5′-CTGAAGAAAGGCAATCCATC-3′
	Reverse primer 5′-GTAGGCTACTACAGGTCATTTG-3′
Human SNGH1	Forward primer 5′-GTGGATTTACGCGCAACGTTG-3′
	Reverse primer 5′-CCAGTAAGCTCTTGTGGGACTG-3′
Human XIST	Forward primer 5′-CTCCAGATAGCTGGCTAACC-3′
	Reverse primer 5′-AGCTCCTCGGACAGCTGCTAA-3′
Human IGF2BP1	Forward primer 5′-GATTAGGCAAGGCTCACACTCAT-3′
	Reverse primer 5′-CACCAAGACAGGTCCTTTCATCC-3′
Human IGF2BP2	Forward primer 5′-AGAGAAGCCTGTCACCCATCCA-3′
	Reverse primer 5′-TCAGTCTTCCAAGCCAAGCCATT-3′
Human IGF2BP3	Forward primer 5′-CCAAGCTAGACAAAGCACTAGAC-3′
	Reverse primer 5′-GCGGCCATTTCATGCAGGGA-3′
Human PUM2	Forward primer 5′-CAACAGCAGCCAATGCACTAATC-3′
	Reverse primer 5′-GCAGCACCCAAAGACGTTACCA-3′
Human ZC3H7B	Forward primer 5′-TCTTCACCTTCCTCTTGCGAGAT-3′
	Reverse primer 5′-GCACCAGGCACTTGGTTGTTG-3′
Human Lin28A	Forward primer 5′-AGGTGCTACAACTAGTGGAGGT-3′
	Reverse primer 5′-GGGTAGGGCTGTGCGATTTCTTC-3′
Human TNRC6A	Forward primer 5′-CAGAACAGATAAAGACCCAGTGT-3′
	Reverse primer 5′-CTGTAGCTCGCTTCGGCATTATTA-3′
Human HNPNPC	Forward primer 5′-CGGAGATGTACGCGGTCAGTAAC-3′
	Reverse primer 5′-GCAATAGGAGGAGTGAGGAGGTA-3′
Human HuR	Forward primer 5′-TTGGGCGGTATCTATCAACTCG-3′
(ELAVL)	Reverse primer 5′-TCAAACCGGATCAAAACGCAACC-3′
Human TIAL	Forward primer 5′-TGGTTGGGTGTGTCGTCAAATC-3′
	Reverse primer 5′-CAGACGCAATTGCCTCCACAGT-3′
Human Runx2	Forward primer 5′-CGAATGGCTAGCACGCTATTAA-3′
	Reverse primer 5′-GTCGCCATAACAGATTCATCCA-3′
Human OCN	Forward primer 5′-TAGTGAAGAAGACCCAGGCGCT-3′
	Reverse primer 5′-ATAGGCCTTCCTGAAAGCCGA-3′
Human ALP	Forward primer 5′-CCACGTCGTTCACATTTGGTG-3′
	Reverse primer 5′-AGACTGCCGCCTGGTAGTTGT-3′
Human U6	Forward primer 5′-TGGAACGCTTCACGAATTTGCG-3′
	Reverse primer 5′-AGACTGCCGCCTGGTAGTTGT-3′
Human GAPDH	Forward primer 5′-TCATGGGTTGTGAACCATGAGAA-3′
	Reverse primer 5′-GGCATGGAACTGTGGTCATGAG-3′

### Bioinformatics analysis

StarBase (version 2.0, http://starbase.sysu.edu.cn/mirlncRNA.php) was used to predict the interactions between miR-21 and its putative lncRNA targets, as well as between TUG1 and its potential RBPs. In addition, binding sites between target RBP and TUG1 transcript were analyzed by CLIP and gene co-expression analysis. Additionally, RBP mapping motifs database (http://rbpmap.technion.ac.il/) was also used for predicting binding domains existed within Lin28A and TUG1.

### Isolation of nuclear and cytoplasmic RNA

The nuclear and cytoplasmic fractions of PDLSCs was separated with an Ambion® PARIS™ Kit (Life Technologies, Carlsbad, CA, USA) based on the manufacturer’s instructions. Approximately 4.0 × 106 cells were lysed in ice-cold cell fractionation buffer and the cytoplasmic fraction was separated from the pellet, containing the nuclear fraction, by aspiration. The pellet was then lysed in cell disruption buffer. Each fraction was mixed with an equal volume of 2× lysis/binding solution, loaded onto a filter cartridges, washed with the wash solution and finally eluted with elution solution (preheated to 99 °C) to obtain the corresponding RNA. The RNA samples were evaluated by qRT-PCR as described above. The relative level of each target gene was determined using the CT method with GAPDH and U6 as the controls for the cytoplasmic and nuclear fractions, respectively.

### Cell transfection

TUG1 knockdown was conducted via lentiviral transfection. Briefly, four lentiviral constructs, designated sh-TUG1-1# to sh-TUG1-4#, were generated based on different regions of the human TUG1 sequence (NCBI accession NR_002323.2). The same lentiviral vector containing an insert of nonspecific RNA oligonucleotide, denoted as sh-NC, was used as a negative control. PDLSCs were transfected with each lentiviral construct at an optimized multiplicity of infection (MOI) of 20. Cells were visualized under a fluorescence microscope and an inverted phase contrast microscope (TH4-200, Olympus, Japan).

For Lin28A knockdown, four siRNA oligonucleotides that complemented with different regions of human Lin28A (ENSG00000131914) were designed and synthesized by Oligobio (Beijing, China). Transfection of PDLSCs were with each of the above siRNA oligonucleotides was performed with the riboFECTTM CP Reagent (Ribobio, Guangzhou, China) at an optimized MOI of 50. For the negative control, PDLSCs were transfected with a nonspecific RNA oligonucleotide. In both experiments, qRT-PCR analysis of TUG1 or Lin28A was performed to evaluate the knockdown efficiency.

The sequences of sh-NC, si-NC, as well as various sh-TUG1 and si-Lin28A constructs were summarized in Tables 2 and 4, respectively.

### Western blotting

Cells were collected, washed with ice-cold PBS, lysed using RIPA reagent containing 1% PMSF, and centrifuged at 12,000×*g* for 5 min. The protein samples were separated on a 12% sodium dodecyl sulfate polyacrylamide gel electrophoresis gel and transferred to a 0.2 µm polyvinylidene fluoride membrane (Millipore, Billerica, MA, USA), which was then blocked with 5% milk for 2 h. The choice and dilution factor of antibodies used in western blotting were described as follows: primary antibodies—rabbit anti-Lin28A (1:500, 16177-1-AP, Proteintech, Chicago, IN, USA) and rabbit anti-GAPDH (1:2000, 10494-1-AP, Proteintech); secondary antibody-goat anti-rabbit horseradish peroxidase-labeled (1:20,000, ZB-2301, ZSJQ-bio, Beijing, China). The blocked membrane was first incubated with the primary antibodies of choice overnight, washed with TBST buffer, and then incubated with the secondary antibodies for 2 h. Protein–antibody complexed were visualized by ECL chromogenic substrate (Millipore) and quantified by densitometry using ImageJ (National Institutes of Health, USA) with GAPDH as control.

### Statistical analysis

All experiments were performed in triplicate unless specified otherwise in the figure legends. Results were expressed as mean ± standard deviation. Statistical analyses were performed by using the one-way analysis of variance or Student’s *t*-test (paired *t-*test) with SPSS (version17.0, IBM, USA). *P* < 0.05 was considered statistically significant.

## Electronic supplementary material

supplementary material 1

supplementary material 2.1

supplementary material 2.4

supplementary material 2.2

supplementary material 2.3

supplementary material3
