# Correction to: Long noncoding RNA TUG1 facilitates osteogenic differentiation of periodontal ligament stem cells via interacting with Lin28A

**DOI:** 10.1038/s41419-018-0750-3

**Published:** 2018-06-15

**Authors:** Qin He, Shuangyan Yang, Xiuge Gu, Mengying Li, Chunling Wang, Fulan Wei

**Affiliations:** 1 Department of Orthodontics, School of Stomatology, Shandong University, Jinan, People’s Republic of China; 2 Shandong Provincial Key Laboratory of Oral Tissue Regeneration, School of Stomatology, Shandong University, Jinan, People’s Republic of China

**Correction to:** *Cell Death Disease* **9**, 455 (2018); 10.1038/s41419-018-0484-2; published online 19 April 2018.

Since publication of this article, the authors found a mistake in the drawing Fig. 5e. After careful checking of all original data, the authors discovered that they had submitted the wrong composite Fig. 5e. The correct Fig. 5 is included below.

**Fig. 5:**
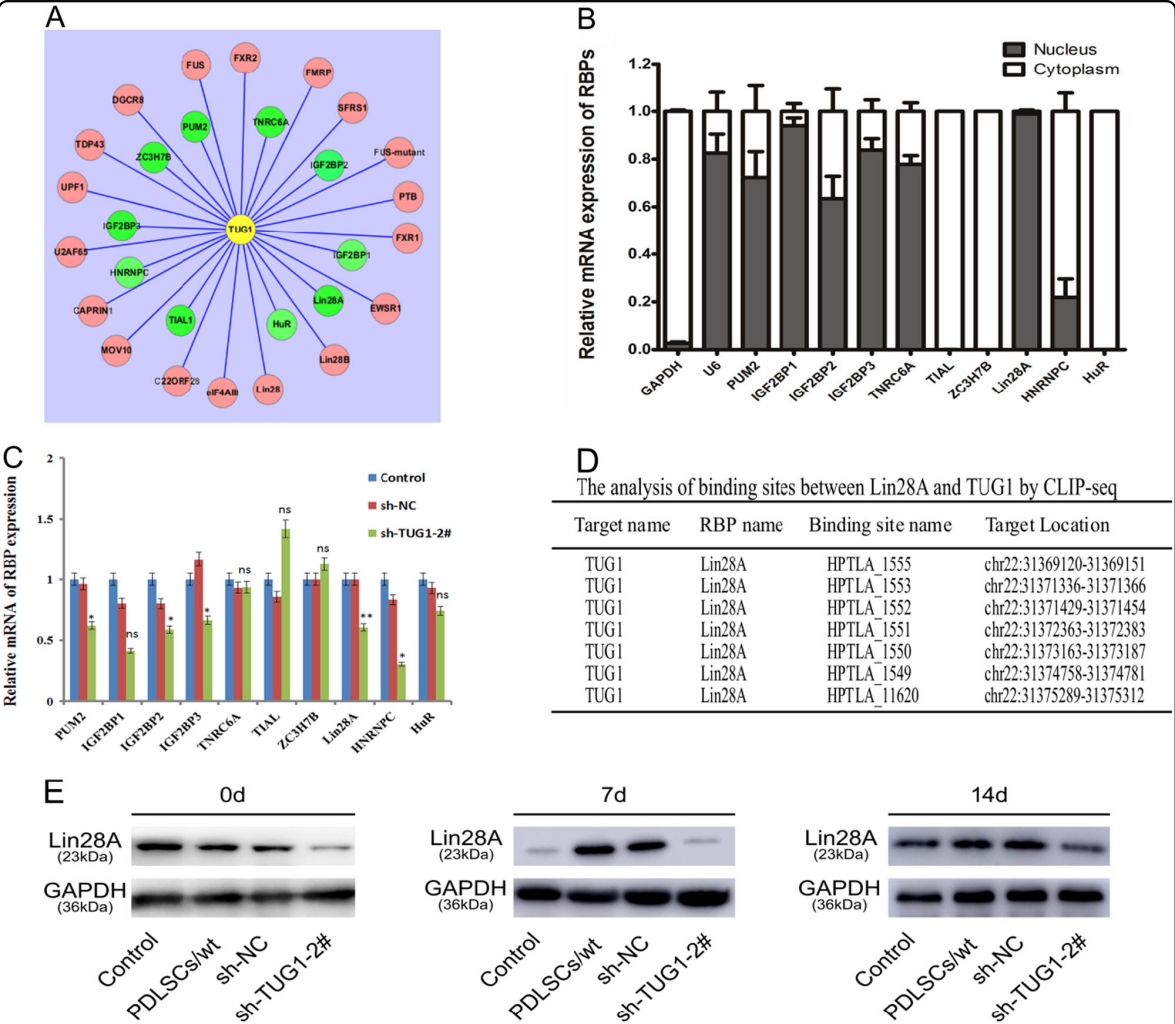
Identification and validation of potential RBPs of TUG1. **a** An interaction network map showing 28 putative RBP candidates that could potentially bind to TUG1. A total of ten candidates were selected for further validation based on literature search, which were shown in green. **b** Subcellular localization of ten RBPs in TUG1 knockdown PDLSCs as determined by qRT-PCR measurement of nuclear and cytoplasmic RNA. GAPDH is the positive control for cytoplasm and U6 is the positive control for nucleus. **c** qRT-PCR analysis of the gene expression levels for the selected RBPs in TUG1 knockdown PDLSCs. **d** Summary of putative binding sites on Lin28A for TUG1 based on results generated from gene co-expression network and CLIP analysis. **e** Western blotting analysis of Lin28A levels in the four above mentioned experiment groups at day 0, 7, and 14 after the osteogenic induction. All experiments were performed in triplicate and results were expressed as means ±SD. *P < 0.05; **P < 0.01; NS not significant

The authors apologise for any inconvenience caused.

