# Supplementary material for: Long noncoding RNA TUG1 facilitates osteogenic differentiation of periodontal ligament stem cells via interacting with Lin28A
Source: Cell Death Dis. 2018 Apr 19;9(5):455. doi: 10.1038/s41419-018-0484-2 (PMC5908786; doi:10.1038/s41419-018-0484-2)
Supplement: Supplementary file 1 — supplementary material 1 [file 41419_2018_484_MOESM1_ESM.docx]

The relationship between TUG1 (lncRNA) and lin28A (RBP)

(The information from rbp mapping database)

Predictions for job: 1500607580

Calculation parameters:

Genome: Human (hg38)

Selected motifs: LIN28A(Hs/Mm):hggagwa

Stringency level: Medium

Conservation filter: Off

**********************************************

ENST00000540687.5|ENSG00000253352.8|OTTHUMG00000030444.4|OTTHUMT00000431950.1|TUG1-003|TUG1|2744|

==============================================================================

Protein: LIN28A(Hs/Mm)

Sequence Position Motif K-mer Z-score P-value

599 hggagwa cagagaa 2.676 3.73e-03

612 hggagwa uggagaa 2.041 2.06e-02

707 hggagwa agcagaa 2.135 1.64e-02

718 hggagwa gggaaaa 2.000 2.27e-02

*******************************************************************************

ENST00000566220.1|ENSG00000253352.8|OTTHUMG00000030444.4|OTTHUMT00000075332.3|TUG1-001|TUG1|1237|

==============================================================================

Protein: LIN28A(Hs/Mm)

Sequence Position Motif K-mer Z-score P-value

228 hggagwa cagagaa 2.676 3.73e-03

241 hggagwa uggagaa 2.041 2.06e-02

336 hggagwa agcagaa 2.135 1.64e-02

347 hggagwa gggaaaa 2.000 2.27e-02

*******************************************************************************

ENST00000519077.3|ENSG00000253352.8|OTTHUMG00000030444.4|OTTHUMT00000431951.2|TUG1-006|TUG1|5673|

==============================================================================

Protein: LIN28A(Hs/Mm)

Sequence Position Motif K-mer Z-score P-value

210 hggagwa cagagaa 2.676 3.73e-03

223 hggagwa uggagaa 2.041 2.06e-02

318 hggagwa agcagaa 2.135 1.64e-02

329 hggagwa gggaaaa 2.000 2.27e-02

4889 hggagwa uggagga 1.784 3.72e-02

4892 hggagwa aggagaa 2.149 1.58e-02

4984 hggagwa aggggaa 2.905 1.84e-03

5006 hggagwa uggagag 2.405 8.09e-03

ENST00000521091.6|ENSG00000253352.8|OTTHUMG00000030444.4|OTTHUMT00000158310.2|TUG1-002|TUG1|2110|

==============================================================================

Protein: LIN28A(Hs/Mm)

Sequence Position Motif K-mer Z-score P-value

28 hggagwa gggaaaa 2.000 2.27e-02

*******************************************************************************

ENST00000602393.1|ENSG00000253352.8|OTTHUMG00000030444.4|OTTHUMT00000467406.1|TUG1-008|TUG1|2233|

==============================================================================

Protein: LIN28A(Hs/Mm)

Sequence Position Motif K-mer Z-score P-value

44 hggagwa aggagaa 1.824 3.41e-02

*******************************************************************************
