# Supplementary material for: Long noncoding RNA TUG1 facilitates osteogenic differentiation of periodontal ligament stem cells via interacting with Lin28A
Source: Cell Death Dis. 2018 Apr 19;9(5):455. doi: 10.1038/s41419-018-0484-2 (PMC5908786; doi:10.1038/s41419-018-0484-2)
Supplement: Supplementary file 2 — supplementary material 2.1 [file 41419_2018_484_MOESM2_ESM.docx]

Title

Interaction between ENST00000519077 (ncRNA) TUG1 and ENST00000326279 (mRNA)Lin28A

Binding site structure predicted by IntaRNA

GGAAGATTGGGAATTAGTCTAAACAGGAAATGGTGGTACACAGAGGCTAGGAGAGGC

...[[[[[[..[[[..........[[[[[..[[[[[[....[[[[[[[[[[.[[[..

ACTCCCTGATGTTTCTTAAAACCACCTCTTCCTATTTTCAGTCTGTGGTTTGGACAG

.]]]]]]]..]]]]]]....]]]]]]..]]]]]]]]..]]]]]].............

Joint secondary structure predicted by RactIP

GGAAGATTGGGAATTAGTCTAAACAGGAAATGGTGGTACACAGAGGCTAGGAGAGGC

...[[[[[[[[[[[..........[[[[[..[[[[[[....[[[[[[[[[[.[[[..

ACTCCCTGATGTTTCTTAAAACCACCTCTTCCTATTTTCAGTCTGTGGTTTGGACAG

.]]]]]]]..]]]]]]....]]]]]]..]]]]]]]]]]]]]]]].............
