# Supplementary material for: Long noncoding RNA TUG1 facilitates osteogenic differentiation of periodontal ligament stem cells via interacting with Lin28A
Source: Cell Death Dis. 2018 Apr 19;9(5):455. doi: 10.1038/s41419-018-0484-2 (PMC5908786; doi:10.1038/s41419-018-0484-2)

The relationship between TUG1 (lncRNA) and lin28A (RBP)

(The information from gene-co-expression analysis)

1

TUG1 ：taurine up-regulated 1 (non-protein coding) [ Homo sapiens (human) ]

Gene ID: 55000

Official Symbol：TUG1provided by HGNC

Official Full Name ：taurine up-regulated 1 (non-protein coding)

Gene type ：ncRNA

Also known as ：TI-227H; LINC00080; NCRNA00080

LIN28A： lin-28 homolog A [ Homo sapiens (human) ]

Gene ID: 79727

Official Symbol ：LIN28A

Official Full Name ：lin-28 homolog Aprovided by HGNC

Ensembl: ENSG00000131914 MIM:611043; Vega:OTTHUMG00000003550

Gene type ：protein coding

Also known as ：CSDD1; LIN28; LIN-28; ZCCHC1; lin-28A

Summary ：This gene encodes a LIN-28 family RNA-binding protein that acts as a regulator of genes involved in developmental timing and self-renewal in embryonic stem cells. The encoded protein functions through direct interaction with target mRNAs and by disrupting the maturation of certain miRNAs involved in embryonic development. This protein prevents the terminal processing of the LET7 family of microRNAs which are major regulators of cellular growth and differentiation. Aberrant expression of this gene is associated with cancer progression in multiple tissues.

2

Binding site structure


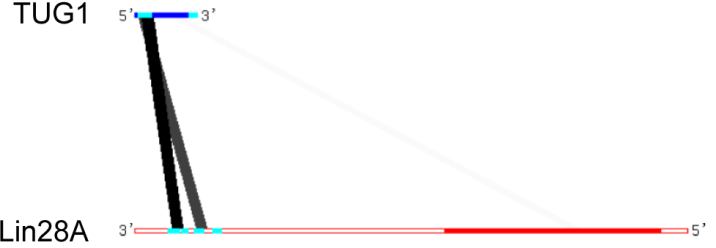


3

The result of relationship (Position of the interaction site，Location of the interaction site，Interaction energy，，Joint secondary structure) between TUG1 and Lin28A


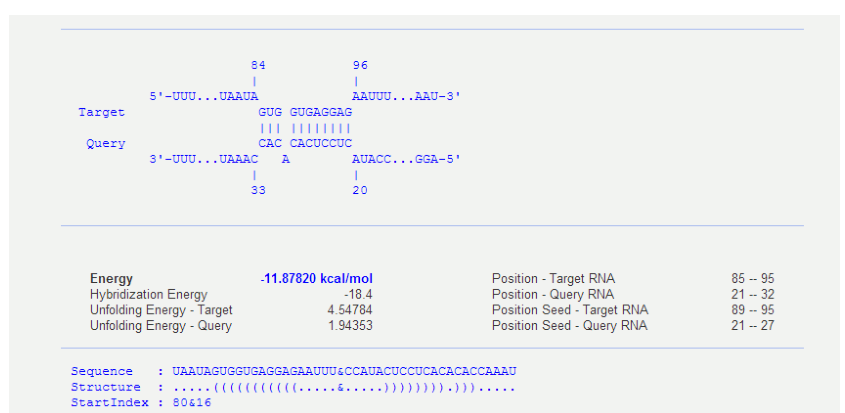


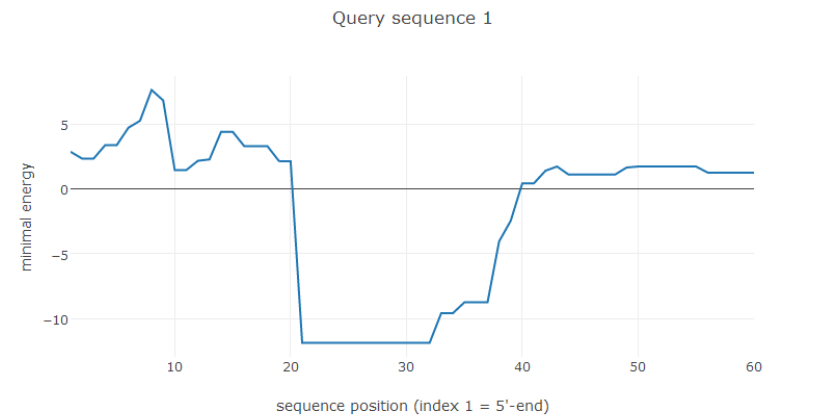


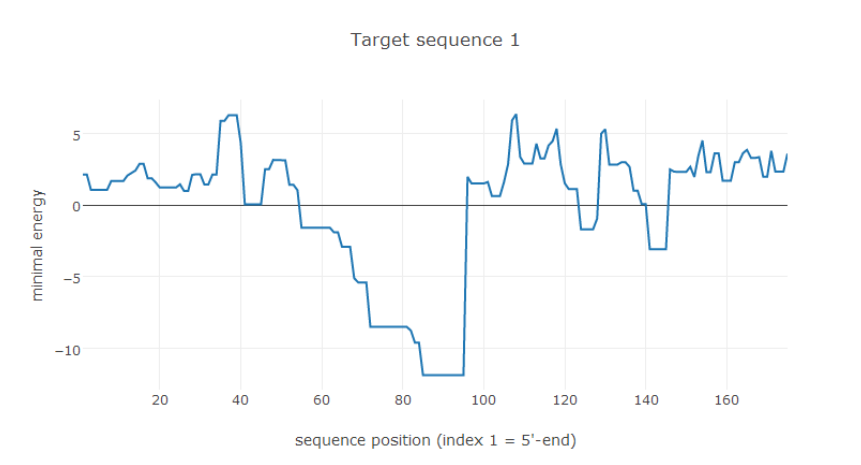


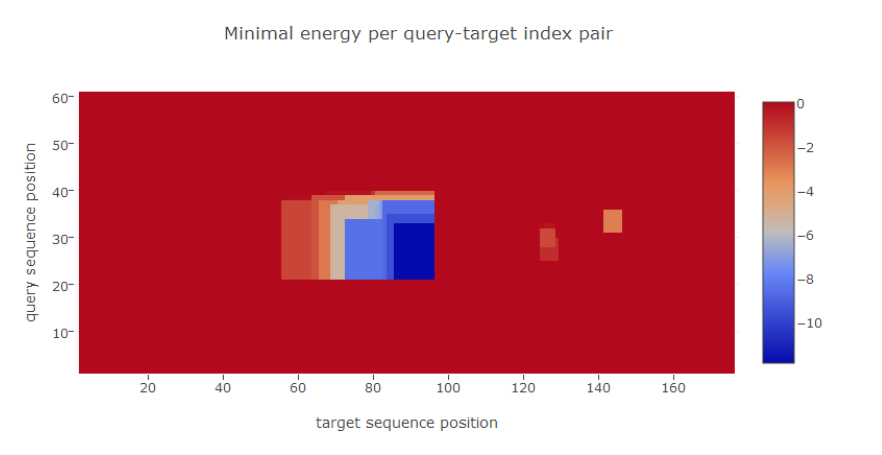


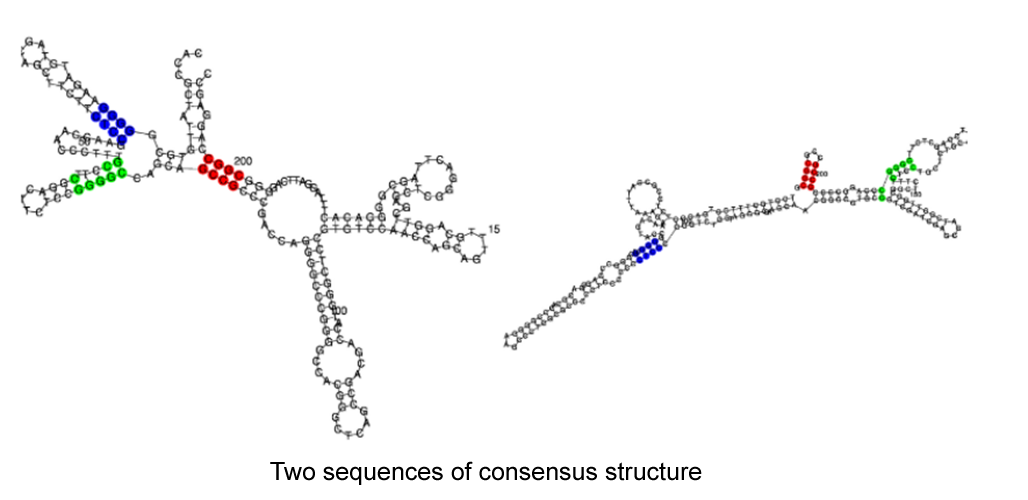


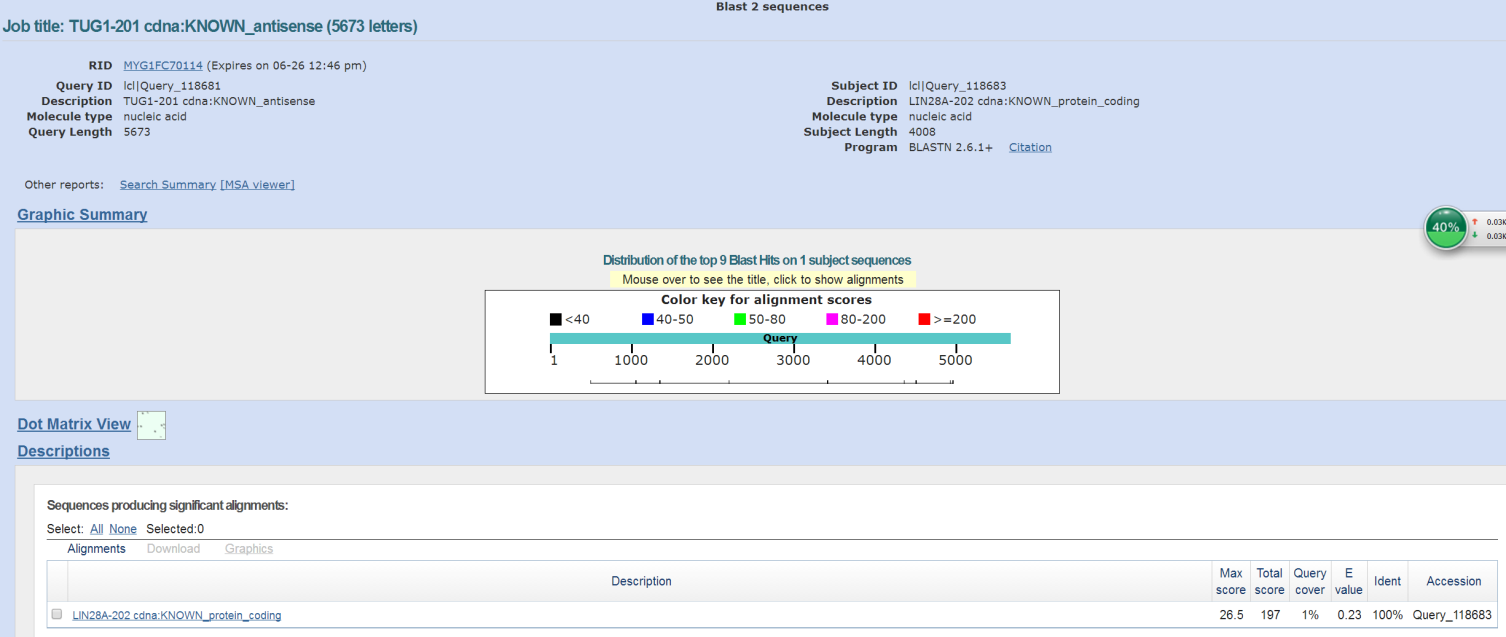


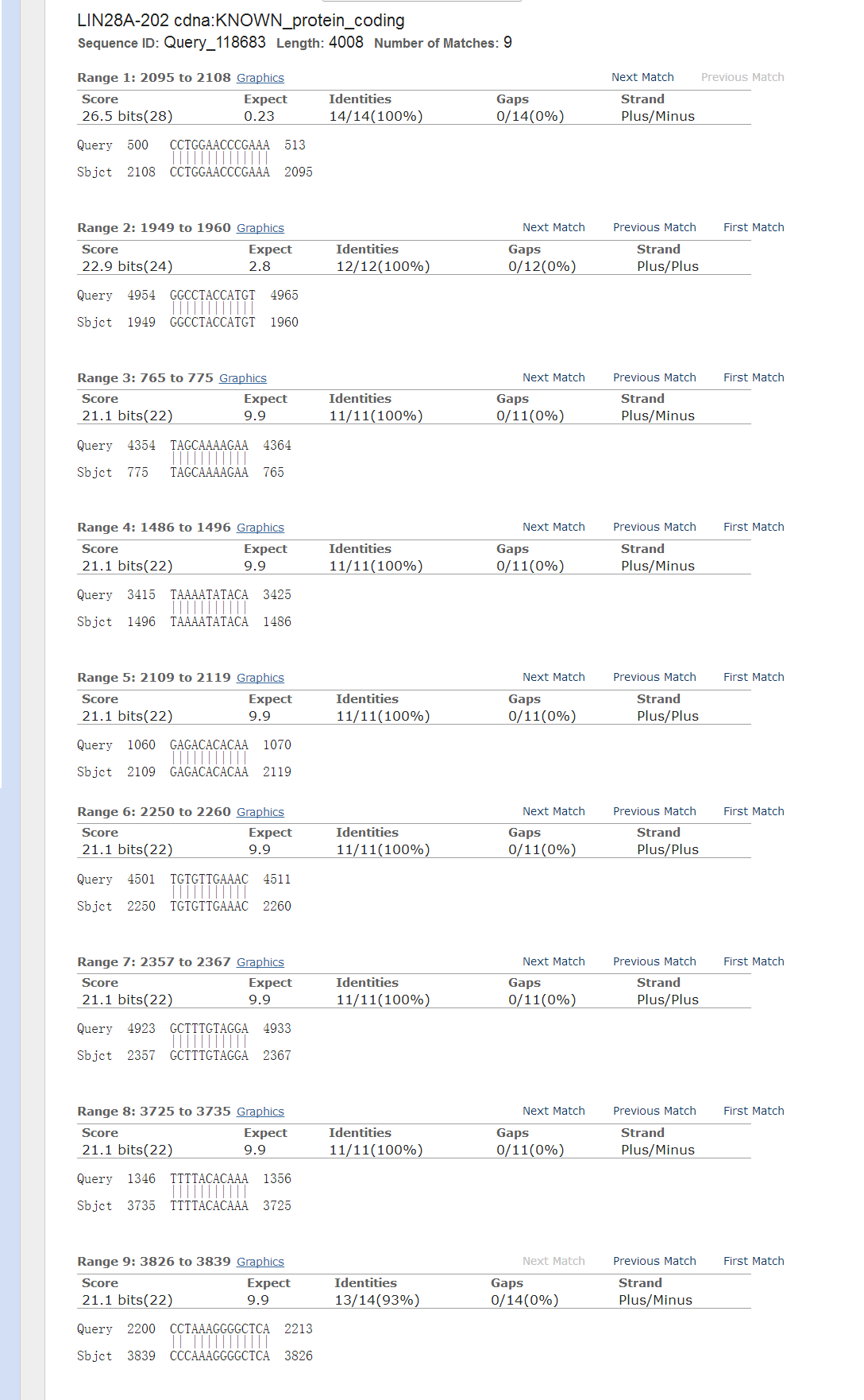

Supplement: Supplementary file 3 — supplementary material 2.4 [file 41419_2018_484_MOESM3_ESM.docx]
