# Supplementary material for: Long noncoding RNA TUG1 facilitates osteogenic differentiation of periodontal ligament stem cells via interacting with Lin28A
Source: Cell Death Dis. 2018 Apr 19;9(5):455. doi: 10.1038/s41419-018-0484-2 (PMC5908786; doi:10.1038/s41419-018-0484-2)
Supplement: Supplementary file 4 — supplementary material 2.2 [file 41419_2018_484_MOESM4_ESM.pdf]

## Interaction between ENST00000519077(ncRNA) and ENST00000326279(mRNA)

## Genomic coordinates of transcripts

ENST00000519077

ENST00000326279

## Interaction energy bewteen these transcripts

| | |
|---|---|
| SumEnergy | -3775.24 kcal/mol |
| MinEnergy | -22.26 kcal/mol |

## Informtion of the interaction site with MinEnergy

### Position of the interaction site on transcripts

| | |
|---|---|
| ENST00000519077 | 4759-4815 |
| ENST00000326279 | 1780-1836 |

### Location of the interaction site on mRNA

| | |
|---|---|
| 5'-UTR | |
| start codon | |
| CDS | |

| | |
|---|---|
| stop codon | |
| 3'-UTR | ✓ |

## Binding site structure predicted by IntaRNA

| |
|---|
| GGAAGATTGGGAATTAGTCTAAACAGGAAATGGTGGTACACAGAGGCTAGGAGAGGC<br>...[[[[[[..[[[..........[[[[[..[[[[[[....[[[[[[[[[[.[[[.. |
| ACTCCCTGATGTTTCTTAAAACCACCTCTTCCTATTTTCAGTCTGTGGTTTGGACAG<br>.]]]]]]]..]]]]]]....]]]]]]..]]]]]]]]..]]]]]]............. |

## Joint secondary structure predicted by RactIP

| |
|---|
| GGAAGATTGGGAATTAGTCTAAACAGGAAATGGTGGTACACAGAGGCTAGGAGAGGC<br>...[[[[[[[[[[[..........[[[[[..[[[[[[....[[[[[[[[[[.[[[.. |
| ACTCCCTGATGTTTCTTAAAACCACCTCTTCCTATTTTCAGTCTGTGGTTTGGACAG<br>.]]]]]]]..]]]]]]....]]]]]]..]]]]]]]]]]]]]]]]............. |
